# Pulmonary lymphangitic carcinomatosis from squamous cell carcinoma of the cervix

**DOI:** 10.1186/1477-7819-8-107

**Published:** 2010-12-03

**Authors:** Rani Kanthan, Jenna-Lynn B Senger, Dana Diudea

**Affiliations:** 1 Department of Pathology and Laboratory Medicine, University of Saskatchewan, Saskatoon SK, Canada

## Abstract

**Introduction:**

Pulmonary metastasis presenting as lymphangitic carcinomatosis arising from squamous cell carcinoma (SCC) of the cervix is a rare event. Poorly represented in the literature, this event is associated with a) difficulty in accurate diagnosis, b) grave prognosis, and the c) lack of recognized predisposing risk factors.

**Case Report:**

A 50 year-old female presented at our practice with a three-month history of a productive cough associated with dyspnoea and shortness of breath. A chest x-ray and computed tomography (CT) scan revealed multiple bilateral patchy areas with subsegmental atelectasis in both lungs which was investigated with a bronchoscopy, left thoracoscopy, and a left lung biopsy. Pathological examination of the wedge biopsy of the left upper lobe revealed neoplastic sheets of cell disturbed along the septal vessels, perivascular/peribronchial lymphatics, and the subpleural lymphatics. This lymphangitic carcinomatosis was confirmed to be metastatic from SCC of the cervix that had been diagnosed and treated two years ago. She was treated with systemic Carbo/Taxol chemotherapy and corticosteroids as a palliative measure. Despite temporary improvement, she died 13 months later.

**Conclusion:**

Pulmonary lymphangitic carcinomatosis is a rare manifestation of metastatic SCC of the cervix. As clinical presentations including radiographic imaging mimics other pulmonary entities, accurate diagnosis remains a challenge. Increased clinical awareness of such patterns of metastases in cervical cancer supported by accurate pathological diagnosis is imperative to guide appropriate therapy in these patients.

## Introduction

Despite advances in screening, cervical cancer remains a significant cause of mortality and morbidity [1]. Cancer of the cervix often metastasizes to nearby organs, and extrapelvic spread is rare. Pulmonary metastasis from carcinoma of the uterine cervix, though uncommon, has been reported in 2.2-9.1% of all cervical cancers [2]. The mean detection interval of pulmonary metastases from the initiation of primary treatment ranges from 2 to 46 months [2]. Histologically, patients with adenocarcinoma and undifferentiated carcinoma of the cervix have higher incidences of pulmonary metastases [2]. Lymphangitic carcinomatosis (LC) secondary to carcinoma of the cervix is exceedingly rare and associated with a grave prognosis with limited survival from 17 days to 24 months [3]. The common primary carcinomas associated with LC are breast, larynx, prostate, thyroid, gallbladder, stomach, and pancreas [3,4]. To the best of our knowledge, this lesion has not been reported in the published English literature for the past five years (last report September 2004) [3].

We report the case of a pulmonary LC metastatic from squamous cell carcinoma (SCC) of the cervix that presented two years following the initial diagnosis of invasive nonkeratinizing SCC of the cervix treated by external beam radiotherapy and intracavitary brachytherapy.

## Case Report

A 48 year-old female presented with vaginal bleeding and abnormal Pap smears. Upon diagnosis of invasive non-keratinizing SCC of the cervix, she underwent a radical hysterectomy with salpingo-oophorectomy which demonstrated positive spread to the pelvic lymph nodes and the parametrium. Pathological examination revealed that the tumour also extensively involved the lower uterine segment. 5 months after this surgery, the woman underwent external beam radiotherapy and intracavitary brachytherapy.

**Figure 1:**
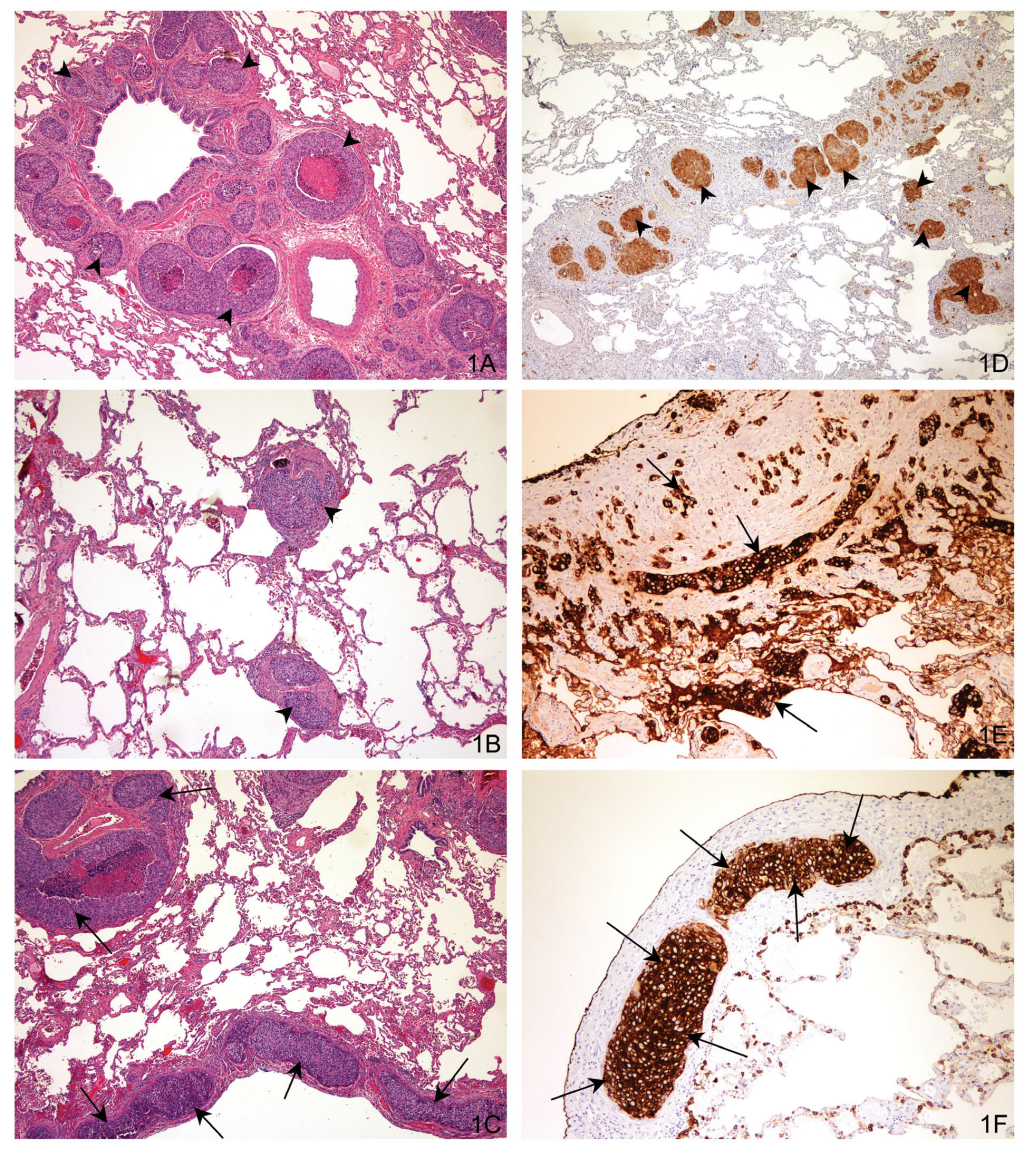
**Histopathology-Lung Biopsy:Haematoxylin and Eosin stained slides [1A,1B and 1C]; Immunohistochemical stained slides [1D, 1E and 1F]**. **1A, 1B, 1C**: Hemotoxylin-eosin stain slides at medium power (magnification ×250) demonstrates the neoplastic cells in a perivascular peribronchial lymphatics (1A, black arrow head), along the septal vessels (1B, black arrow head), and subpleural lymphatics (1C, black arrow). **1D, 1E, 1F**: Immunohistochemicals stain at medium power (magnification ×250): p16 shows strong positive nuclear and cytoplasmic staining of the peribronchial lymphatics (1D, black arrow head); high molecular weight keratin shows strong positive membranous staining of the lesional cells (1E, black arrow); cytokerain 7 (CK7) demonstrates the strong positive staining of the lesional cells in the subpleural lymphatics (1F, black arrow).

Two years later, the patient presented with a three-month history of a productive cough, shortness of breath, and a 2-3 week history of progressive exertional dyspnea. X-rays of the chest demonstrated a reticular nodular pattern, and CT scans revealed multiple bilateral patchy areas of ground glass opacity scarring with focal areas of subsegmental atelectasis within both lungs. A differential diagnosis included interstitial pneumonia versus non-cardiogenic edema. The woman underwent a bronchoscopy, left thoracoscopy, and an open wedge left lung biopsy. Pathological examination of the left lung biopsy confirmed the presence of neoplastic sheets of cells classically distributed along the septal vessels, perivascular, peribronchial, and subpleural lymphatics. Subpleural nodules were also identified with the presence of neoplastic cells distending the subpleural lymphatics confirming LC (Figures 1A, 1B, 1C). On immunohistochemical analysis, the lesional cells were strongly positive to p16 (Figure 1D), high and low molecular weight keratins (Figure 1E), cytokeratin-7 (CK7) (Figure 1F), CK19, and pan keratin, and negative to CK20, p63, and EGFR. Based on these findings, she was diagnosed to have lymphangitic carcinomatosis in the lung metastatic from SCC of the cervix.

She was started on chemotherapy (Carbo/Taxol) with corticosteroids while in the hospital, and was discharged ten days later. Post-treatment improvement of clinical symptoms was paralleled by radiographic imaging that showed marked interval improvement of the nodular opacifications and the interstitial thickening that had previously been noted. Despite this improvement, she subsequently died 13 months after the initial diagnosis.

## Discussion

Cervical cancer most frequently spreads by direct extension to the surrounding tissue such as the vagina, uterus, and pelvic cavity [5]. Such local recurrences are usually diagnosed two to three years after initial treatment [6]. Metastases to distant, extrapelvic sites such as the lungs, para-aortic lymph nodes, and bones occur in the advanced stages of the cancer [2,7]. Metastases to the lung comprise up to 3% of cervical cancer treatment failures in stage IA, 15% in stage IB, 20-25% in stage IIB, and 40% in stage IIIB [6]. The incidence of pulmonary metastasis differs depending on the histological type of cervical cancer with an increased risk associated in patients with adenocarcinoma, anaplastic cancer, and small cell neuroendocrine tumours. Metastasis from SCC of the cervix is less common and usually does not surpass 5% [6]. Such metastases to the lung may take many forms: solitary and multiple parenchymal nodules, lymphangitic carcinomatosis, tumour emboli, endobronchial metastasis, and pleural effusion [8]. However, it is important to confirm that this SCC is indeed a metastatic lesion to the lung and not a primary pulmonary SCC. In this context, p16 is a useful marker for the discrimination between cervical and pulmonary SCC as overexpression of p16 has been consistently observed in HPV-related cervical cancer [9]. Pulmonary metastasectomy for solitary metastasis to the lung is a safe and acceptable treatment to improve survival in cases where there is adequate control of the primary tumour without extrapulmonary metastasis [10].

Lymphangitic carcinomatosis (LC) is the diffuse infiltration and obstruction of the parenchymal lymphatic channels by a tumour [11]. Cervical cancer rarely metastasizes to the lung, and the presentation of lymphangitic carcinomatosis is a rare pathological diagnosis. In a review of 245 cases of thoracic metastases from cervical carcinoma by Sostman and Matthay, lymphangitic pattern of metastasis was not observed [12]. SCC of the cervix metastasizing to the lung as LC represents a very uncommon occurrence in the published English literature. A PubMed/Medline search using the key words "lymphangitic carcinomatosis" and "cervix/cervical" yielded 9 results, with the most recent written by Storck et al in 2004 [3]. In LC, tumour spread through the lymphatic system is hypothesized to occur in one of two ways: either through hematogenous spread to the interstitial space followed by the lymphatics or in a retrograde manner from the lymph node to the periphery [8]. Incidence of LC accounts for 6-8% of all metastatic diseases to the thorax. The common carcinomas associated with LC are breast, larynx, prostate, thyroid, gallbladder, stomach, and pancreas [3,4]. This type of cancerous spread generally infiltrates bilaterally in both the subpleural and interstitial lymphatics [13]. Some theorize that immunosuppression be a risk factor for LC development, whereas others suggest that tumour cell proliferation in the hilar nodes cause lymphatic flow obstruction, leading to retrograde spread of the tumour cells through the pulmonary lymphatics [3]. It is postulated that as patients with cervical cancer now live longer and die less frequently from local disease or obstructive uropathy due to improved radiation therapy and chemotherapy, the occurrence of metastases to distant sites such as the lung may be observed more frequently in the distant future.

Clinical manifestations of LC such as dyspnea and non-productive cough often lead to the incorrect diagnosis of pneumonia, pneumonitis, pulmonary embolism, congestive heart failure, asthma, and sarcoidosis [3]. Lymphangitic spread of cervical cancer represents a state of advanced metastatic disease with a grave prognosis and shortened survival [5]. Radiographic imaging does not provide the specificity required for diagnosing LC, as LC may have seemingly normal chest radiographs or other nonspecific reticular-nodular patterns with lymphadenopathy and pleural effusions [4]. A computed tomography (CT) scan may support the diagnosis by detecting the 'beaded chain' appearance due to uneven thickening in the septa of the lung secondary to lymphatic vessels, demonstrating cell infiltration [3,4]. Bronchoscopy with washings and sputum cytology are both unreliable in accurately confirming the diagnosis of LC [3]. A transbronchial or an open lung biopsy is often required for a definitive pathological diagnosis of pulmonary lymphangitic carcinomatosis due to the proximity of lymphatics to the peribronchial space [8].

LC has a poor prognosis as it indicates advanced metastatic disease [5]. Often, extensive involvement of the hilar, mesenteric, and para-aortic nodes, diaphragmatic, liver, and abdominal metastases can also co-exist in these patients [13]. The use of radiation treatment is a point of dispute, as it has been argued it may disrupt the immunologic surveillance mechanisms and physical barrier created by the nodes, thereby promoting metastases to the lungs [5]. Complete response to chemotherapy is rare; however, patients who receive platinum-based chemotherapy with corticosteroids do show some degree of improvement [3]. Combination chemotherapy such as cisplatin + paclitaxel or topotecan has demonstrated improvement in a) response rates, b) progression free survival (PFS), and c) sustained quality of life (QOL) assessments [14,15]. Regardless of treatment, however, survival of patients with LC is short. Nevertheless, accurate diagnosis of metastatic SCC to the lung and LC is important in avoiding any unnecessary and potentially harmful treatments [5].

## Conclusions

Pulmonary lymphangitic carcinomatosis is a rare manifestation of metastatic SCC of the cervix and is associated with a poor prognosis. Clinical presentations of LC including radiographic imaging mimic other pulmonary diseases as diagnostic pitfalls. Despite the lack of recognized predisposing risk factors and the difficulty in accurate diagnosis, recognition of metastatic SCC to the lung and LC is significant as it is associated with a grave prognosis. Increased clinical awareness of such patterns of metastases in cervical cancer supported by accurate pathological diagnosis is necessary to guide appropriate therapy in these patients.

## Consent/Competing interests

Publication of these cases without patients consent was exempted by the Research ethics board of University of Saskatchewan as the consent of the patient or her next of kin for publication could not be obtained. A copy of the waiver of consent from the Research Ethics Office, University of Saskatchewan, has been submitted to the Editor-in-Chief of this journal.

The authors declare that they have no competing interests.

## Authors' contributions

RK is the corresponding and first author of this manuscript. JLS is a summer student who contributed to the acquisition, analysis, and interpretation of data. DD has made substantial contributions to the conception and design of this manuscript. All authors read and approved the final manuscript.
